# Impact of Yangtze River Water Transfer on the Water Quality of the Lixia River Watershed, China

**DOI:** 10.1371/journal.pone.0119720

**Published:** 2015-04-02

**Authors:** Xiaoxue Ma, Lachun Wang, Hao Wu, Na Li, Lei Ma, Chunfen Zeng, Yi Zhou, Jun Yang

**Affiliations:** 1 School of Geographic and Oceanographic Sciences, Nanjing University, Nanjing, China; 2 Hydrology and Water Resources Investigation Bureau of Jiang Su Province, Nanjing, China; 3 Flood Control and Drought Relief Command Center of Jiangsu Province, Nanjing, China; NERC Centre for Ecology & Hydrology, UNITED KINGDOM

## Abstract

To improve water quality and reduce the negative impacts of sudden inputs of water pollution in the Lixia River watershed, China, a series of experimental water transfers from the Yangtze River to the Lixia River were conducted from 2 December 2006 to 7 January 2007. Water samples were collected every six days at 55 monitoring sites during this period. Eight water parameters (water temperature, pH, dissolved oxygen (DO), chemical oxygen demand (COD), potassium permanganate index (COD_Mn_), ammonia nitrogen (NH_4_^+^-N), electrical conductivity (EC), and water transparency (WT)) were analyzed to determine changes in nutrient concentrations during water transfers. The comprehensive pollution index (Pi) and single-factor (Si) evaluation methods were applied to evaluate spatio-temporal patterns of water quality during water transfers. Water quality parameters displayed different spatial and temporal distribution patterns within the watershed. Water quality was improved significantly by the water transfers, especially for sites closer to water intake points. The degree of improvement is positively related to rates of transfer inflow and drainage outflow. The effects differed for different water quality parameters at each site and at different water transfer times. There were notable decreases in NH_4_^+^-N, DO, COD, and COD_Mn_ across the entire watershed. However, positive effects on EC and pH were not observed. It is concluded that freshwater transfers from the Yangtze River can be used as an emergency measure to flush pollutants from the Lixia River watershed. Improved understanding of the effects of water transfers on water quality can help the development and implementation of effective strategies to improve water quality within this watershed.

## Introduction

Water transfer engineering has been used successfully in many water bodies for irrigation, water supply, flood control, water quality improvement, power generation, and so on [1,2]. There are presently 13 large-scale water transfer projects in China. However, they are mainly used to resolve water shortages caused by uneven distribution of water resources, such as the South-to-North water diversion project and the Luanhe River Diversion project. Water transfer from the Yangtze River to Lake Taihu is only the project used to improve water quality and to reduce the risk of algal blooms in Lake Taihu. Water transfer is the most effective emergency countermeasure to enhance water exchange, dilute polluted water, and improve water quality, by transporting freshwater from a comparatively clean source to a more polluted water body, and has the advantages of low cost, easy implementation, and quick response [1,3]. Previous studies have examined the effects of water transfer on donor and receiving ecosystems, on biological variables, and in mitigating eutrophication, alleviating water crises, and ensuring water security [4–6]. However, Fornarelli and Antenucci found that few studies have concentrated on the effects of water transfer on nutrient concentration [7]. Hu et al. showed notable positive effects on debasing the concentration of phytoplankton, total nitrogen, and dissolved oxygen in some sub-areas [3]. Hu et al. [1] and Zhai et al. [5] demonstrated that water transfer could lower phytoplankton concentrations. However, Yao et al. showed that water- sediment regulation in the Yellow River has no significant effect on the variation in dissolved inorganic nutrient concentrations [8]. Hu et al. reported that large-scale water transfer resulted in less pronounced improvement in water quality [1]. Li et al. also showed that the effects of water transfer on overall water quality improvement were spatially and temporally heterogeneous [9]. In conclusion, the impacts of water transfer on water quality remain a challenge deserving of study. A literature review showed that few previous studies have focused on the effects of water transfer on overall water quality improvement in plain (low topography) river networks, and so the effectiveness of water transfer in such systems remains largely unknown.

In this study, water transfer is defined as the transfer of large volumes of freshwater from the Yangtze River to the relatively polluted Lixia River watershed to dilute pollutants and facilitate water purification. When the quality of inflow water from the “supplier” is comparatively better than that of the “receiver,” water transfer plays an important role in improving water quality [10]. Therefore, water quality in the Lixia and Yangtze rivers should first be compared in order to determine whether the Yangtze River offers potential to improve water quality. Liu et al. argue that water quality in the Lixia River is poor, with 98% of their samples exceeding the Type III water quality standard (surface water quality standard in China (GB3838–2002) S1 Table) and 14.1% exceeding the Type V water quality standard [11]. In the Yangtze River, 81.5% of the collected samples comply with water quality standards Types I–III, while water samples from Jiangsu comply with the Type II standard [12]. These findings indicate better water quality in the Yangtze River, such that water transfer offers the prospect of improving the water quality in the Lixia River. Some reaches of the Lixia River watershed act as passages for the eastern route of the South-to-North Water Transfer project, and so the present quality of the Lixia River directly affects the success of the transfer project. The main purpose of this study is to quantify the spatial and temporal effects of experimental water transfers on water chemical parameters and to evaluate the possibility of improving water quality by transferring water from the Yangtze River. This study provides a scientific basis for future project scheduling and operational management.

## Materials and Methods

### Study area

**Fig 1:**
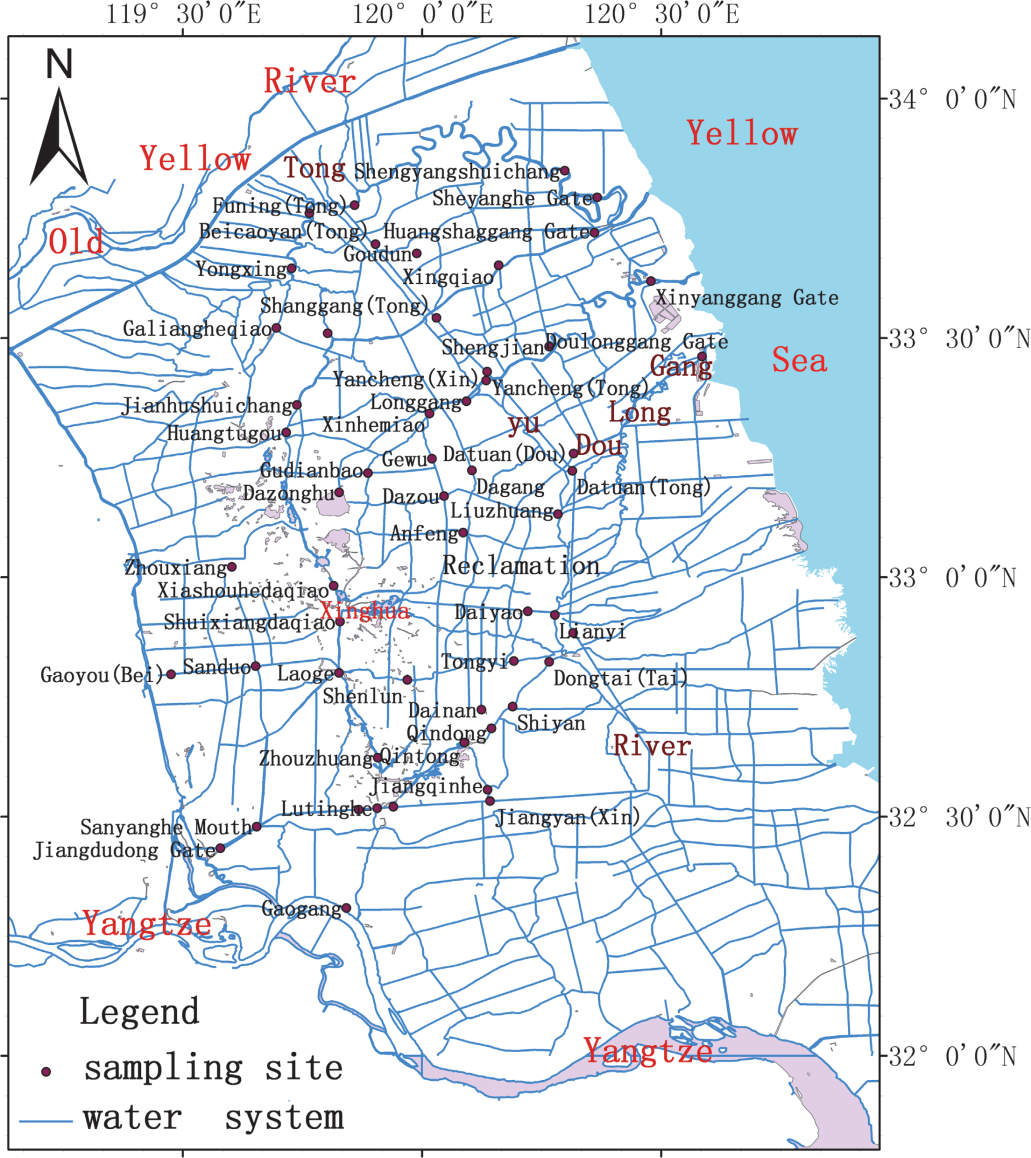
Study area and monitoring sites.

The Lixia River watershed (119̊08’E-120̊56’E, 2̊12’N-34̊10’N) is located in mid-eastern Jiangsu, China, which is north of the Yangtze River, and the basin is drained from the northwest and southwest, toward the northeast into the Yellow Sea. According to the characteristics of the natural landscape and water system, the Lixia River watershed can be divided into the central Lixia River Basin, the reclamation area of northern Doulong, and the reclamation area of southern Doulong, bounded by the Tongyu River and Doulong Harbor. The central Lixia River watershed is known as the Lixia-River Plain. It is part of the Jianghuai plain, and was formed by long-term accumulation of sediment from the Old Yellow, Yangtze, and Huaihe rivers. The central region of the river basin is a cauldron-shaped depression. This unique geographical environment, coupled with the construction of dikes following the foundation of the People’s Republic of China (PRC), makes the water system relatively occluded and independent. The coastal reclamation area is inclined from southeast to northwest, and ground elevation is lowest in the northern reclamation area. The basic characteristics of the river network, such as low flow velocity, variable flow, poor exchange, and weak self-purification systems, make the flushing of contamination very difficult. The basin’s cauldron-shape allows rainwater to quickly flow from surrounding areas to the center of the watershed, causing rapid increases in water level. This water is then slowly discharged into the Yellow Sea along the waterway after exceeding the flood warning level. The scope of experimental monitoring includes the sources of water transfers, the main aqueduct, the main ocean outfall waterway, the central Lixia River Basin, and the primary water source locations in the urban areas of the watershed. This study selected 55 monitoring points, clustered along various rivers within the Lixia River Basin (see Fig. 1).

### Water transfer measurements

Water transfers from the Yangtze River to the Lixia River watershed were conducted from 3 December 2006 to 7 January 2007, by the Water Resources Department of Jiangsu Province (Hydrology and Water Resources Investigation Bureau of Jiang Su Province; Flood Control and Drought Relief Command Center of Jiangsu Province) and Nanjing University. No specific permissions were required for use of these locations and activities. The field studies did not involve any endangered or protected species. The sampling dates were: 2, 4, 10, 16, 22, and 28 December 2006; and 3, 9, 13, and 17 January 2007. Two major water control projects in Jiangdu and Gaogang controlled the quality and quantity of the incoming water for dilution. Four major coastal channels that discharge to the Yellow Sea (the Sheyang River, Huangshagang River, Xinyanggang River, and Doulonggang River) acted as outflow channels for the Lixia River watershed. Four floodgates were installed at each of the above outflow channels to control the rate of outflow, based on measured water level in the watershed. There was a major rainfall event prior to this test, which led to higher water levels. Therefore, the Lixia River area only required draining at the beginning of the experiment, with water diversion and drainage occurring at the same time following a drop in water level. The Gaogang hub began to pump water from the Yangtze River when the water level dropped to 1.35 m on 8 December 2006, and transferred 118 million m^3^ of water. From December 11 to 12, approximately 0.08 million m^3^ of water was transferred from the Yangtze River, when there was a difference in water level between the two sides of the Jiangdu east gate. However, owing to its lower water level, the Yangtze River could not meet the need for artesian water. Consequently, from 13 December 2006 to 8 January 2007, approximately 311 million m^3^ of water was pumped by the Jiangdu pumping stations. The total amount of water transferred during this experimental water transfer was 437 million m^3^. From 3–10 December 2006, water was discharged through the Sheyang, Huangshagang, Xinyanggang, and Doulonggang river channels, with average daily outflow rates of 312 m^3^/s, 123 m^3^/s, 193 m^3^/s, and 115.2 m^3^/s, respectively. Conversely, from 1–7 January 2007, water was discharged through the Huangshagang, Xinyanggang, and Doulonggang river channels, with average daily outflow rates of 101.3 m^3^/s, 156 m^3^/s, and 90.3 m^3^/s, respectively. The total volume of effluent water transported via the Sheyang, Huangshagang, Xinyanggang, and Doulonggang river sluices was 1.27 billion m^3^.

#### Monitored parameters and chemical analyses

Water samples were collected before the diversion, and then collection was repeated every six days after diverting water from all 55 sites, in order to monitor changes in water quality during the experimental period. The sampling, preservation, and transportation of water samples to laboratory facilities followed standard methods (Technical regulation of water quality sampling SL187–96, Regulation for Water Environmental Monitoring SL219–98) [13–14]. Water temperature (T), pH, dissolved oxygen (DO), electrical conductivity (EC), and water transparency (WT) were measured on-site, whereas chemical oxygen demand (COD), potassium permanganate index (COD_Mn_), and ammonia nitrogen (NH_4_^+^-N) were measured following transfer to the laboratory. The various water quality parameters use differing units and analytical methods, which are summarized in Table 1. The quality of analytical data was ensured through careful standardization, procedural blank measurements, and the use of duplicate samples as in previous studies [15, 16].

**Table 1:** Water quality parameters and their associated abbreviations, units, regulatory standards, and analytical methods.

Variables	Abbreviations	Units	Standard	Analytical methods
Ph	pH	pH unit	GB6920–1986	pH meter
Water temperature	T	°C	GB13195–1991	Mercury thermometer
Water transparency	WT	Cm	SL87–1994	Disc method
Dissolved oxygen	DO	mg/L	GB11913–1989	Probe method
Electrical conductivity	EC	mS/m	SL78–1994	Electrometric
Potassium permanganate index	COD_mn_	mg/L	GB11892–1989	Permanganate method
Chemical oxygen demand	COD	mg/L	GB11914–1989	Dichromate method
Ammonia nitrogen	NH_4_^+^-N	mg/L	GB7479–1987	Nessler's reagent colorimetry

### Comprehensive pollution index evaluation method

All mathematical and statistical computations used Origin 8.0 (OriginLab) software. In this study, water quality was determined via the single-factor evaluation and comprehensive pollution index methods. The comprehensive pollution index method is based on single-factor evaluation, and the single-factor evaluation method is widely used to assess water quality in China according to the National Water Quality Guidelines for Surface Water [17]. These guidelines use the lowest water quality classification of individual indicators to determine the type of comprehensive water quality status. The comprehensive pollution index is an important method of evaluating water quality, and has been applied in many previous studies [18–21]. A pollution index is created to compare variations in spatial distribution and water quality among different water-flushing areas in the Lixia River watershed. We use the following formulae to calculate the surface water pollution index:

$$Si=\frac{Ci}{C_{0}}$$ (1)

$$Pi=\frac{1}{n}\times Si$$ (2)

where *P*_*i*_ is the comprehensive pollution index, *Si* is the single-factor pollution index, *Ci* is the actual concentration of a particular surface water pollutant (mg/L), and *C*_*0*_ is the concentration (mg/L) of a particular pollutant in surface water, according to the water quality standard.

If the only measured chemical parameter is DO, the single-factor pollution index (S_i_) uses the following formula:

$$S_{i}\equiv \left\{ \begin{array}{ll} \;0 & \;C_{i}\geq 8\\ \;1-\frac{C_{i}-C_{0}}{C_{0}} & \;5\leq C_{i}≺8\\ \;1+(C_{i}-C_{0}) & \;0\leq C_{i}≺5 \end{array}\right.$$ (3)

In this study, in order to retain coherence of all three zones, *C*_*0*_ is set as the Type III standard concentration of the Environmental Quality Standards for Surface Water (GB3838–2002).

## Results

### In situ observation of water quality improvement in the Lixia River watershed

Basic statistical analyses (range, mean, and standard deviation) of the eight water quality parameter are summarized in Table 2. Almost all observed pH values ranged from 7.4 to 9, which is within the range of 6 to 9 specified by the surface water guidelines. Water temperatures showed moderately low values (4–13°C). For EC and transparency, no regulation or standard is available in China. Water transparency can be used as an indicator of water quality, with lower transparency indicating higher concentrations of organic elements, suspended matter, and nutrients [22]. EC can indicate water quality in areas unaffected by seawater, with a higher value indicating a greater number of ions in water, which has an adverse effect on water quality [23]. Nitrogen pollution was the most prominent type of contamination: approximately 5% of the samples contained NH_4_^+^-N concentrations that exceeded water quality standard Type V, with 14% exceeding the Type III standard. DO concentrations varied greatly, from 1.2 mg/L to 17.8 mg/L, with 74.8% of the samples complying with Type I. For COD, 33.9% of the samples complied with water quality standard Type III, whereas 15% of the samples exceeded the Type III standard. For COD_Mn_, 69.4% of the samples complied with the Type III standard, while 15% exceeded this level.

**Table 2:** Physico-chemical characteristics of water quality during diversion.

Parameters	N	Mean	Min.	Max.	SD	C_V_(%)
**pH**	550	8.13	7.4	9	0.32	3.9
**T**	550	6.73	2.9	13	2.24	33
**EC (*u*S/cm)**	550	806.67	242	1791	296.14	37
**Transparency**	550	47.78	8	150	21.99	46
**DO (mg/L)**	550	8.93	1.2	17.8	2.19	25
**NH_4_^+^-N (mg/L)**	550	0.74	0.05	4.01	0.61	82
**COD**_**Mn**_ **(mg/L)**	550	4.87	1.8	9.7	1.07	22
**COD (mg/L)**	550	15.1	9	38.5	5.39	36

*NOTE*: *N refers to number of samples*.

Among dissolved oxygen (DO), chemical oxygen demand (COD_Cr_), potassium permanganate index (COD_Mn_), ammonia nitrogen (NH_4_^+^-N), electrical conductivity (EC), and water transparency (WT), the improvements in DO, NH_4_^+^-N, COD_Mn_, and COD_Cr_ were most pronounced. At the beginning of the water transfer, a marked increase in DO concentration (approximately 3.05mg/L) was detected in comparison with the mean values for 2–4 December. DO concentration continued to increase to 9.67mg/L after water transfer ceased. NH_4_^+^-N concentration was reduced by approximately 0.1797mg/L in comparison to the mean values for 2–4 December (0.8256mg/L), and then increased to 0.96mg/L after water transfer ceased. COD_Cr_ concentration declined from 16.1mg/L (pre-transfer values) to 14.89mg/L during water transfer, then declined further to 14.74mg/L after water transfer ceased. COD_Mn_ concentration decreased from 5.24mg/L (pre-transfer values) to 4.69mg/L during water transfer, but then increased to 5.05mg/L after water transfer ceased.

**Fig 2:**
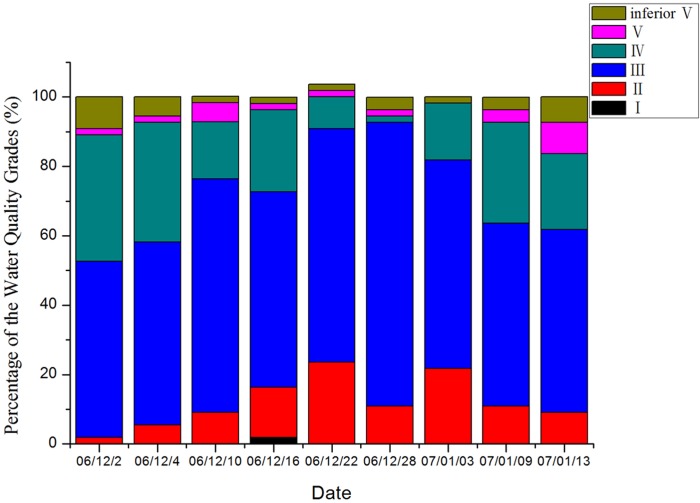
Water quality grades in Lixia River according to sampling date.

Standard water quality (SWQ) was defined as that which met water quality standard Type III, of the Environmental Quality Standards for Surface Water. As can be seen from Fig. 2, the number of samples satisfying the SWQ criteria increased gradually and then decreased, with 92.7% of the samples on 28 December complying with water quality standard Type III or better (also known as the threshold for drinking water). The number of samples satisfying the SWQ on 9 January decreased sharply after water transfer ceased, and after declining to a minimum it then rebounded slightly on 17 January. The drawing of water from the Yangtze River played a positive role in improving the quality of the regional water environment in the Lixia River, with 52.7% to 65% of all samples reaching the established SWQ.

From the findings discussed above, it is concluded that freshwater can effectively flush pollutants from the Lixia River watershed and improve water quality by means of water exchange, dilution, and diversion.

### Effects of inflow/outflow rate on water quality improvement in the Lixia River watershed

**Fig 3:**
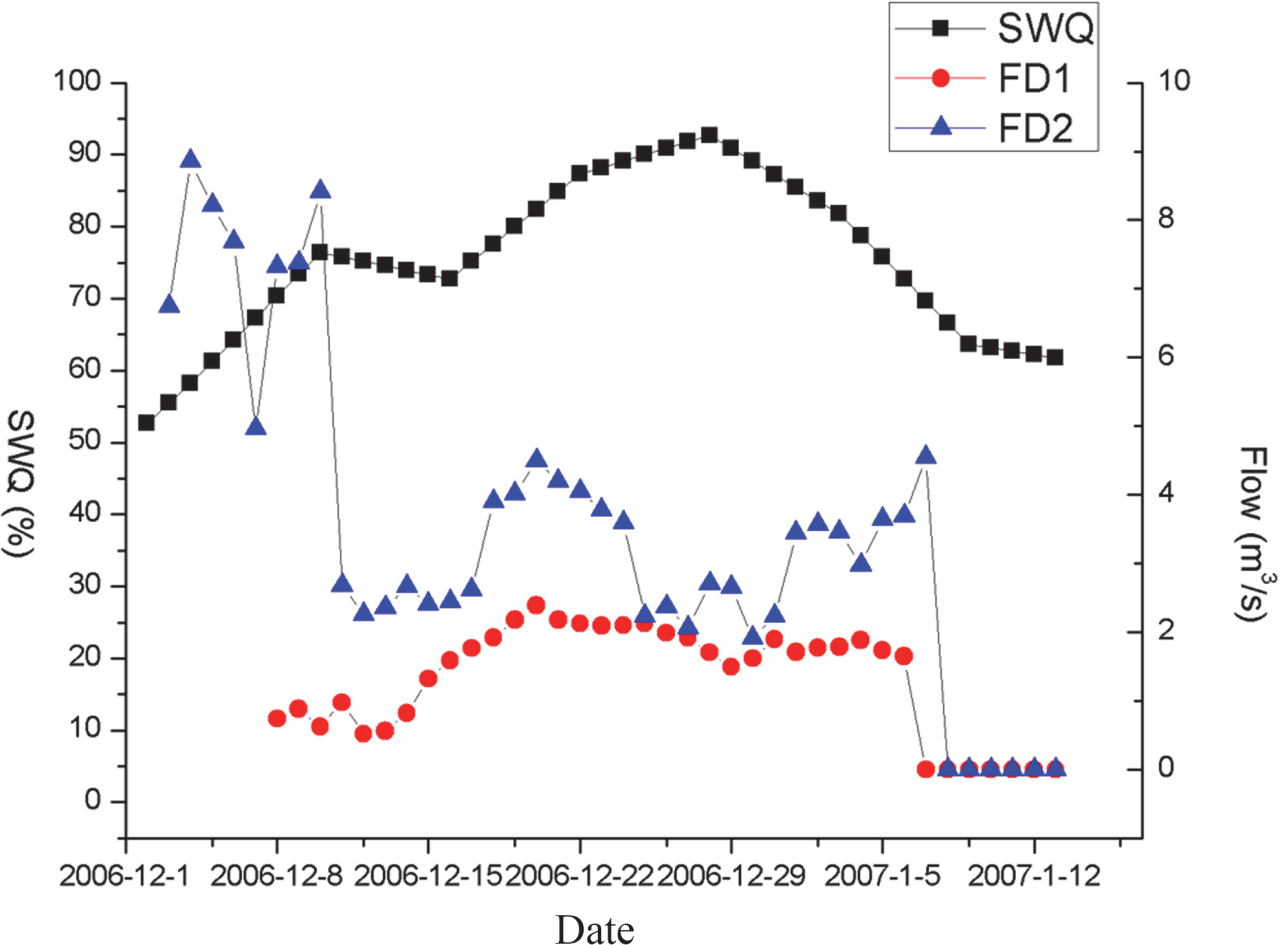
Comparison of inflow (FD1) / outflow (FD2) rates and standard water quality (SWQ).

In order to understand the relationship between water quality improvement and flow rate, the relationship was assessed between SWQ and inflow/outflow transfer rate. The transferred inflow rate (FD1) refers to the sum of the inflow rates of the Gaogang and Jiangdu water gates. The outflow rate (FD2) refers to the sum of the average daily flow rates of the Huangshagang, Doulonggang, Sheyanghe, and Xinyanggang rivers. At the beginning of the water transfer, the SWQ first rose, and then decreased as water outflow decreased (Fig. 3). Subsequently, a remarkable rise in SWQ was detected as water transfer continued. In later periods, SWQ decreased from 92.7% to 65.5% owing to the influence of the water transfer. Owing to the continuous rainfall prior to the experiment, the Lixia River watershed must first drain and then transfer the water in order to maintain the water balance and to avoid the risk of flooding. The hub began to pump water from the Yangtze River when the water level dropped to 1.35 m on 8 December 2006. However, water quality still showed a notable increase in the early stages of the experimental water transfer, when there was no water transfer from the Yangtze River (Fig. 3). At low water inflow rate, SWQ decreased as water outflow decreased, and then increased as inflow rate increased. Therefore, as shown by Fig. 3, water quality was improved firstly by the water flow and secondly by the water transfer. In short, SWQ varied according to fluctuations in the inflow/outflow transfer rates.

**Fig 4:**
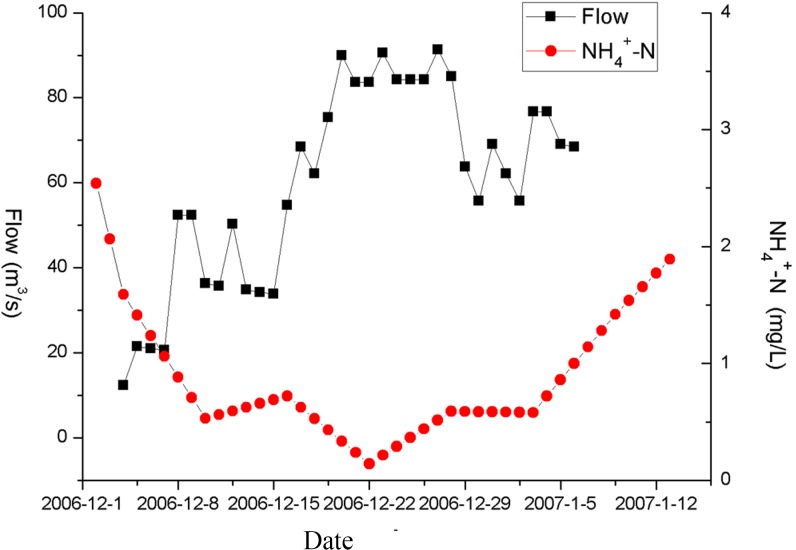
Comparison of NH4+-N concentration and internal water flow in Taidong River.

**Fig 5:**
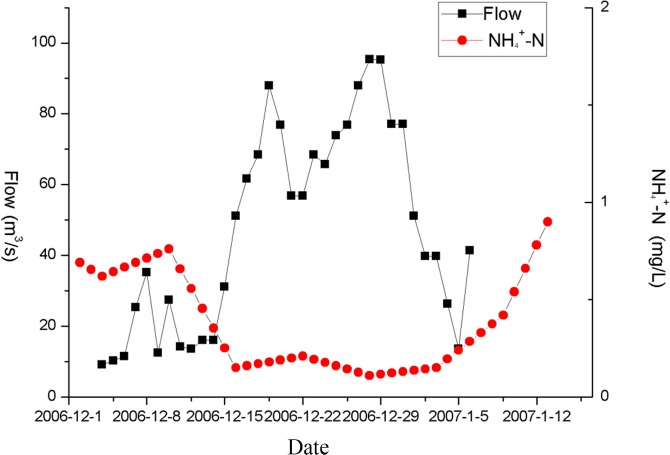
Comparison of NH4+-N concentration and internal water flow in Sanyang River.

The water from the Yangtze River flows a great distance from the Jiangdu and Gaogang water gates to the four coastal rivers. About 36.9% of the transferred water along the Taidong River and 29.1% of the transferred water along the Sanyang River flows into this watershed. Therefore, similarly to the previous assessment of the inflow/outflow transfer rates, the influence of internal water flow is assessed through observation of the ammonia nitrogen concentration in the Taidong and Sanyang rivers. As seen in Fig. 4 and Fig. 5, there is a strong negative correlation between observed ammonia nitrogen concentration and internal water flow. There are two primary factors that can account for this phenomenon: one is that the water flow could enhance water exchange, increasing the rate of pollutant degradation and improving the self-purification capacity of the water; the other is that increasing the quantity of water being transferred could enhance the dilution of polluted water.

### Spatial and temporal variation in water quality during water transfers

The 55 sampling sites showed varying improvements in water quality. Among the six variables DO, COD, COD_Mn_, NH_4_^+^-N, EC, and WT, the greatest improvements were observed in DO, COD, and NH_4_^+^-N. The significant spatial and temporal disparities of these six parameters are outlined below.

**Fig 6:**
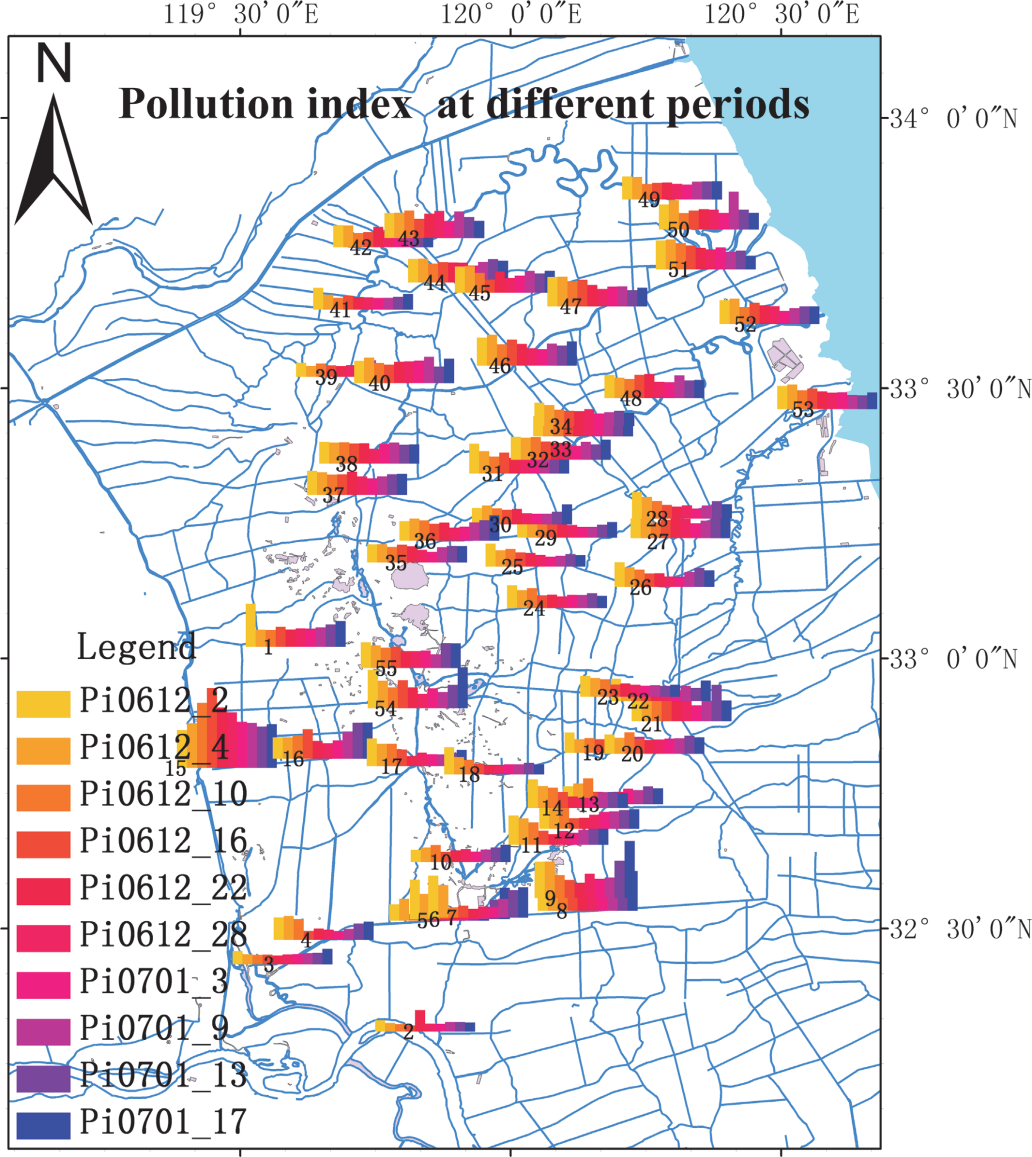
Spatial and temporal variation of water quality.

During water diversion, the decline in NH_4_^+^-N concentration was more pronounced in the Southern Zone, which is close to the diversion port. NH_4_^+^-N concentration near the diversion port began to increase quickly following water transfer. In the southeast, which is farther from the diversion port, water transfer had relatively little effect on NH_4_^+^-N concentration. In the Beichengzi and Sanyang rivers, however, NH_4_^+^-N concentration increased to a certain value before decreasing slowly as the water transfer progressed. This may be due to the presence of dead water regions in the tributaries of these rivers. During the water transfer, the sudden inflow of extraneous water induced backwater from a river branch, causing an increase in pollutant concentrations in the main river. These pollutant levels later decreased as polluted water was diluted and flushed from the river. However, along with the increase in the total volume of transferred water, EC concentrations showed no significant changes at different water transfer times. EC and COD_Mn_ concentrations varied at different sampling points: EC concentrations in offshore areas were higher than those in pelagic regions. The COD_Mn_ concentration in the diversion port was lower than those at other sampling points. The DO concentration of the whole watershed increased slowly during the water transfer, before decreasing quickly after the water transfer ceased. The WT measurements showed significant temporal and spatial variations. During the water transfer, WT increased slowly in offshore areas, whereas those in the diversion port initially decreased and then increased, while WT values at other sampling sites slowly decreased. COD concentrations in the southwest first increased suddenly, and then decreased in comparison to the mean, based on the value for 3 December 2006, while COD concentrations in offshore areas first increased slowly before decreasing. As seen from Fig. 6, a more significant improvement was detected in the southwest, while the deterioration of water quality was most severe in the mid-west. During transfer of water from the Yangtze River to the Lixia River on 3 December 2006, the pollution index decreased gradually at many sampling sites. Among all sites, the sharpest decreases were observed in the Taizhou penstock river (which draws water from the Yangtze River), Xintongyang Canal, Taidong River, and LuTing River (located nearest the Yangtze River) (Fig. 6). By the end of the study, the pollution index had increased gradually in many sampling sites, with higher values correlating with the length of time during which water transfers ceased. Sewage was prevented from flowing into the main river stem during diversion, owing to the higher water level. The water level was reduced after the water transfers ended, leading to a decrease in water quality. Water quality did not stabilize until the water level stopped declining. The pollution index in the Beichengzi River (Fig. 6, sampling site 5) initially increased and then decreased, indicating that water quality in the Beichengzi River first deteriorated before improving. This is mainly due to the stirring of sewage in stagnant water pools, resulting from the inflow of water, causing sewage to subsequently flow into the main river. Therefore, this initially led to contamination of the river before water quality subsequently improved (Table 3).

**Table 3:** Sampling sites, sampling site codes, and river locations.

Code	River	Site	Code	River	Site
1	Sanyang River	Zhouxiang	29	Ganggou River	Dagang
2	Taizhou penstock River	Gaogang Gate	30	Dongwo River	Gewu
3	Xintongyang canal	Jiangdu Gate	31	Xiyan River	Xinhemiao
4	Sanyang River	Sanyang River	32	Mengshe River	Longgang
5	Taizhou penstock river	Yinjianghe mouth	33	Xinyang gang	Yancheng (xin)
6	Xintongyang canal	Lutinghe mouth	34	Tongyu River	Yangcheng (tong)
7	Xintongyang canal	Taidonghe mouth	35	Mengshe River	Dazong hu
8	Xintongyang canal	Jiangyan (xin)	36	Zhuli Gou	Gudianbao
9	Jiangqin River	Jiangqin River	37	Xitang River	Huangtugou (xi)
10	Luting river	Zhouzhuang	38	Xitang River	Jianhushuichang
11	Taidong River	Qintong	39	Galiang River	Galiang bridge
12	Taidong River	Qindong	40	Xitang River	Jianhu
13	Taidong River	Shiyan	41	Sheyang River	Yongxing
14	Yanjing River	Dainan	42	Sheyang River	Funingshuichang
15	Beichengzi River	Gaoyou (north)	43	Sheyang River	Funing (she)
16	Beichengzi River	Sanduo	44	Tongyu River	Beicaoyan (tong)
17	Beichengzi River	Laoge	45	Haihe	Goudun (hai)
18	Bengyan River	Shenlun	46	Tongyu River	Shanggang (tong)
19	Bengyan River	Tongyi	47	Huangsha gang	Xinqiao
20	Taidong River	Dongtai (tai)	48	Xinyang gang	Shengjian
21	Tongyu River	Dongtai (tong)	49	Sheyang River	Sheyangshuichang
22	Chelu River	Lianyi	50	Sheyang River	Sheyang Gate
23	Chelu River	Daiyao	51	Huangsha gang	Huangshagang Gate
24	Haigou river	Anfeng	52	Xinyang gang	Xinyanggang Gate
25	Xingyanjie River	Dazou	53	Doulong gang	Doulonggang Gate
26	Xingyanjie River	Liuzhuang (xing)	54	Shangguan river	Shuixiangqiao
27	Tongyu River	Datuan (tong)	55	Xiaguan River	Xiaguanheqiao
28	Doulong gang	Datuan (dou)			

## Discussion

The effects of water transfer on overall improvement in water quality were spatially and temporally heterogeneous. Only certain areas showed improvements in water quality during the early water transfer periods, owing to the uneven distribution of the water transfer in the Lixia River watershed. The subareas that displayed negative environmental effects were the Beichengzi River, the Tongyu River, and the Northwest Zone in the initial days following the introduction of transferred water to the watershed. For example, the clearly observed increases in COD_Mn_ and COD concentration in the Beichengzi River were unexpected. However, the water transfer displayed more positive effects in the southwest and northeast zones. For example, there were clear decreases in COD_Mn_ and COD concentration in the Taizhou penstock River in this early period. Samples collected from the inflow point located in the Taizhou penstock river and the Xintongyang Canal showed clear improvements in water quality. At sampling sites located far from the water inflow point, some degree of transportation lag is evident before such areas display a significant dilution and diversion of pollutants in the north of the watershed. The locations of input water inflow and outflow points in the Lixia River influence the effectiveness of water transfers by affecting the hydrodynamic processes and flushing rates in different subzones (Fig. 6). Welch [24] and Hilt et al. [25] deemed that improvement of water quality is more significant in small watersheds than that in large watersheds. Hu et al. [1] deemed that the effectiveness of water transfer is generally limited by size, water source, water transportation path and time, and inflow/outflow rates. Li et al. showed that improvement of water quality was dependent on hydrodynamic conditions induced by flow rates and wind directions and magnitudes [9]. Therefore, not all of the basin subzones experienced a notable reduction in nutrient concentration during the initial water transfers.

The local current field or flow rates near the inlet and outlet points are considered key factors in the effectiveness of water transfer. Previous studies showed that simulation by means of an ecological model is appropriate for assessing the effects of water transfers. Li et al. set the optimal transferred inflow rate to maximize the improvement of the watershed’s water transfer rates at minimum economic cost and environmental impact, based on a three-dimensional Environmental Fluid Dynamic Code (EFDC) model [26]. However, Hu et al. showed that the models over-estimated the improvements in water quality observed in practice [1]. The ecological models could not simulate real-world scenarios with complete accuracy, owing to the selection of limited model parameters and data; the present study therefore utilizes direct comparison to measure water quality improvement. The results indicate that a greater improvement in water quality is generally associated with higher transfer flow rates (Fig. 3 and Fig. 4). However, this transfer of water occurs only once. Although an increased transferred flow rate can improve water quality, it is difficult to optimize the transfer flow and timing to maximize the improvements provided by water exchange. In order to determine the optimal transfer flow rate, we can attempt to set a specific flow rate before ceasing water transfers as nutrient concentrations decrease. Then, as nutrient concentrations increase to levels observed prior to the water transfer, another water transfer can be initiated at a different flow rate. However, this method may be costly and time-consuming. Therefore, it may be of more value to consider the combination of indirect simulation and direct comparisons in future studies.

The source of transferred water is considered another key factor in the effectiveness of the transfer. The extent of water quality improvement in river networks is limited by the quality and quantity of the water being transferred. Excessive transfer of water will waste water resources and result in flooding. Conversely, insufficient transfer of water will not produce any notable improvement in water quality. Welch et al. suggested that 50% of lake inputs might result in a noticeable effect on water quality [27]. During the water transfer, we did not consider the amount of water needed to improve water quality, which is a variable that requires further study. As influent water often contained comparatively higher nutrient concentrations in the Yangtze River compared to the input lake itself, Hu et al. [1,3] and Zhai et al. [5] demonstrated that water transfer could decrease the concentration of phytoplankton, but argued that intensive water transfers over long periods could accelerate eutrophication in areas of Lake Taihu where nutrient concentrations are lower than those of the influent water. Li et al. suggested that water transfers might actually be a major pollutant source for parts of the lake [9]. It is possible that some exiting pollutants in the Yangtze River might be flushed into the watershed, resulting in localized areas of poor water quality [26]. This nutrient overloading is a controversial issue for the Yangtze River Transfer project. Here, we discuss nutrient load based on monitoring data from the water resources center in Jiangsu, China. High loadings of COD and NH_4_^+^-N can be calculated from the Yangtze River data. The results show that COD in the Lixia River watershed external input load is 3,837 tonnes, while NH_4_^+^-N loading is 57 tonnes. The results also show that the additional nutrient loads from the sewage entering the water body comprises 7,175 tonnes for COD and 426 tonnes for NH_4_^+^-N. As a result of dilution and diversion, nutrient concentrations should decrease during the water transfer and within a certain timeframe. However, across longer time spans, the nutrient concentrations will increase as pollutants discharge from sewage outlets upon termination of the water transfer. Combined with the calculated transferred outflow and nutrient loads from the Lixia River watershed to the Yellow Sea, it is concluded that the water transfer resulted in net decreases of 7,680 tonnes COD load and 587,000 tonnes NH_4_^+^-N. The water transfer resulted in evident improvement in water quality within many subzones of the Lixia River watershed, with the assistance of water exchange, dilution, and diversion. Thus, water transfers can only be used as an emergency measure in order to improve water quality and reduce the effects of sudden instances of water pollution.

## Conclusions

This study investigated the temporal and spatial distributions of water quality changes throughout the Lixia River watershed, by evaluating the effects of experimental water transfers from the Yangtze River on major water quality parameters (e.g., COD, COD_Mn_, DO, NH_4_^+^-N, etc.). The results show notable improvements in water quality, suggesting that these transfers can be used to improve water quality in the Lixia River watershed. The magnitude of water quality improvement was dependent on the quality of transferred water and associated flow rates, as well as subzones and fluctuations in transfer duration. Among the eight water quality parameters, the decrease in NH_4_^+^-N and COD concentrations, and the increase in DO concentration were more pronounced during the water transfer. The transferred water flow rate can accelerate water exchange and water dilution. At subareas far from the Yangtze River, changes in water quality lagged behind changes in water inflow rate. Following the water transfer, there was a lag before any improvement in water quality was observed throughout the entire watershed, with a longer duration of water transfer resulting in further improvements in water quality. Once the water transfer ceased, nutrient concentrations increased to levels lower than those observed prior to the water transfer. Thus, the transfer of river water could provide a useful emergency measure for improving water quality. The results of this study can help local governments and decision makers better understand the effects of water transfer projects on water quality and encourage improved management of these projects.

## Supporting Information

S1 Table Environmental quality standards for surface water (mg/L). (DOCX)
