# Supplementary material for: Impact of Yangtze River Water Transfer on the Water Quality of the Lixia River Watershed, China
Source: PLoS One. 2015 Apr 2;10(4):e0119720. doi: 10.1371/journal.pone.0119720 (PMC4383563; doi:10.1371/journal.pone.0119720)
Supplement: S1 Table — (DOCX) [file pone.0119720.s001.docx]

There exists many water quality standards, such as whose concerned with surface water, ground water, and sea water, each of which have their own classification standard. The classification standard used for surface water quality is utilized in this study. This surface water quality standard is an authorized guideline now available in China ([GB3838-2002](#_ENREF_22)), in which water quality standard Type I refers to water quality comparable to the source water or national nature reserve water, water Types II and III refers to the protection zones mainly used in the centralized surface drinking water source, Types IV and V represent water that is no longer fit for drinking but still suitable for industrial and agricultural usage, and water quality worse than Type V is severely detrimental to aquatic ecosystem health ([GB3838 2002a](#_ENREF_22)).

Table S1

Environmental quality standards for surface water(mg/L) ([GB3838 2002a](#_ENREF_22))

| number | parameters |  | TypeⅠ | TypeⅡ | Type Ⅲ | Type Ⅳ | Type Ⅴ |
| --- | --- | --- | --- | --- | --- | --- | --- |
| 1 | Water  Temperature  (℃) |  | Man-made water temperature change should be limited :  Average weekly maximum temperature rise≤1  Average weekly maximum temperature drop ≤2 | | | | |
| 2 | pH |  | 6---9 | | | | |
| 3 | DO | ≥ | saturation ratio 90%（or7.5） | 6 | 5 | 3 | 2 |
| 4 | COD_Mn_ | ≤ | 2 | 4 | 6 | 10 | 15 |
| 5 | COD_Cr_ | ≤ | 15 | 15 | 20 | 30 | 40 |
| 6 | BOD_5_ | ≤ | 3 | 3 | 4 | 6 | 10 |
| 7 | NH_3_-N | ≤ | 0.15 | 0.5 | 1.0 | 1.5 | 2.0 |
| 8 | TP | ≤ | 0.02 （Lake=0.01） | 0.1 （Lake=0.025） | 0.2 （Lake=0.05） | 0.3 （Lake=0.1） | 0.4 （Lake=0.2） |
| 9 | TN | ≤ | 0.2 | 0.5 | 1.0 | 1.5 | 2.0 |
| 10 | Cu | ≤ | 0.01 | 1.0 | 1.0 | 1.0 | 1.0 |
| 11 | Zn | ≤ | 0.05 | 1.0 | 1.0 | 2.0 | 2.0 |
| 12 | Fˉ | ≤ | 1.0 | 1.0 | 1.0 | 1.5 | 1.5 |
| 13 | Se | ≤ | 0.01 | 0.01 | 0.01 | 0.02 | 0.02 |
| 14 | As | ≤ | 0.05 | 0.05 | 0.05 | 0.1 | 0.1 |
| 15 | Hg | ≤ | 0.00005 | 0.00005 | 0.0001 | 0.001 | 0.001 |
| 16 | Cd | ≤ | 0.001 | 0.005 | 0.005 | 0.005 | 0.01 |
| 17 | Cr^6+^ | ≤ | 0.01 | 0.05 | 0.05 | 0.05 | 0.1 |
| 18 | Pb | ≤ | 0.01 | 0.01 | 0.05 | 0.05 | 0.1 |
| 19 | cyanide | ≤ | 0.005 | 0.05 | 0.2 | 0.2 | 0.2 |
| 20 | volatile phenol | ≤ | 0.002 | 0.002 | 0.005 | 0.01 | 0.1 |
| 21 | petroleum | ≤ | 0.05 | 0.05 | 0.05 | 0.5 | 1.0 |
| 22 | anionic surfactant | ≤ | 0.2 | 0.2 | 0.2 | 0.3 | 0.3 |
| 23 | Sulfide | ≤ | 0.05 | 0.1 | 0.05 | 0.5 | 1.0 |
| 24 | [fecal coliform](http://dict.cn/fecal%20coliform)（unit/L） | ≤ | 200 | 2000 | 10000 | 20000 | 40000 |

*Note: This surface water quality standard is an authorized guideline now available in China (GB3838-2002), in which water quality standard Type I refers to water quality comparable to the source water or national nature reserve water, Types II and III refers to the protection zones mainly used in the centralized surface drinking water source, Types IV and V represent water that is no longer fit for drinking but still suitable for industrial and agricultural usage, and water quality worse than Type V is severely detrimental to aquatic ecosystem health.*
